# Stable transformation of fluorescent proteins into *Nosema bombycis* by electroporation

**DOI:** 10.1186/s13071-022-05236-4

**Published:** 2022-04-21

**Authors:** Zhanqi Dong, Na Gao, Boyuan Deng, Xuhua Huang, Congwu Hu, Peng Chen, Qin Wu, Cheng Lu, Minhui Pan

**Affiliations:** 1 State Key Laboratory of Silkworm Genome Biology, Southwest University, Chongqing, 400716 China; 2 Key Laboratory of Sericultural Biology and Genetic Breeding, Ministry of Agriculture, Southwest University, Chongqing, 400716 China; 3 The General Extension Station of Sericulture Technology of Guangxi Zhuang Autonomous Region, Nanning, 530007 China

**Keywords:** Microsporidia, *Bombyx mori*, *Nosema bombycis*, Electro-transformation

## Abstract

**Background:**

Microsporidia are a group of intracellular parasitic eukaryotes, serious pathogens that cause widespread infection in humans, vertebrates, and invertebrates. Because microsporidia have a thick spore wall structure, the in vitro transformation, cell culture, and genetic operation technology of microsporidia are far behind that of other parasites.

**Methods:**

In this study, according to an analysis of the life-cycle of microsporidia, *Nosema bombycis*, and different electro-transformation conditions, the transduction efficiency of introducing foreign genes into *N. bombycis* was systematically determined.

**Results:**

We analyzed the direct electro-transformation of foreign genes into germinating *N. bombycis* using reporters under the regulation of different characteristic promoters. Furthermore, we systematically determined the efficiency of electro-transformation into *N. bombycis* under different electro-transformation conditions and different developmental stages through an analysis of the whole life-cycle of *N. bombycis*. These results revealed that foreign genes could be effectively introduced through a perforation voltage of 100 V pulsed for 15 ms during the period of *N. bombycis* sporeplasm proliferation.

**Conclusions:**

We present an effective method for electro-transformation of a plasmid encoding a fluorescent protein into *N. bombycis*, which provides new insight for establishing genetic modifications and potential applications in these intracellular parasites.

**Graphical Abstract:**

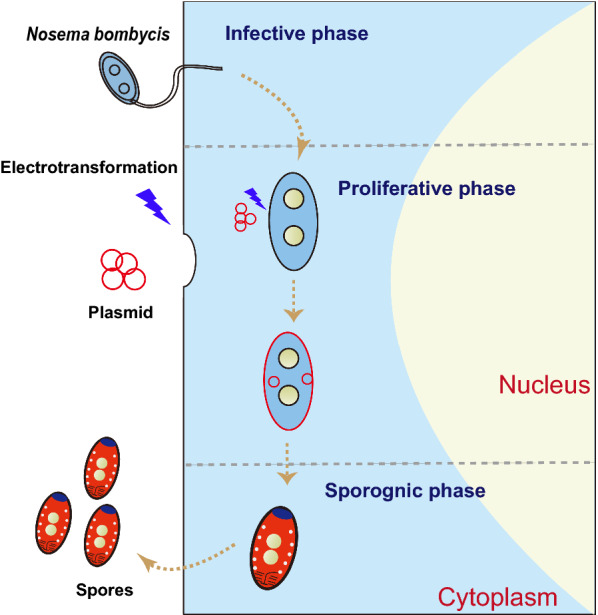

## Background

Microsporidia are a group of obligate intracellular parasitic single-cell eukaryotes with unique biological characteristics. They can infect almost all invertebrates and vertebrates, including humans [1–5]. In patients with immune deficiencies, infection by microsporidia such as *Enterocytozoon bieneusi*, *Encephalitozoon cuniculi*, and *Encephalitozoon intestinalis* can lead to malignant diseases of the respiratory system, urinary system, and skin ulceration, which may be life-threatening [6–8]. *Encephalitozoon cuniculi* infects mice, guinea pigs, and other animals, leading to tissue lesions, brain death, and other symptoms [9–11]. *Nosema bombycis*, *Vairimorpha apis*, and *Vairimorpha ceranae* infect economically important insects, hinder their development, reduce spawning and reproduction, and eventually lead to their death and heavy economic losses [12–15]. The most effective way to control and treat microsporidia is by analyzing microsporidia–host interactions and infection mechanisms [2, 16, 17]. Changing the microsporidian genome through genetic manipulation technology is undoubtedly the most effective means to improve our understanding of the mechanism of microsporidian infection and proliferation mechanisms.

Since the completion of the detailed genome map of *E. cuniculi* in 2001, the genome sequences of *E. bieneusi*, *Trichomonas hominis*, *V. apis*, *V. ceranae*, and *N. bombycis* have been gradually completed, indicating that the microsporidia studies have entered the era of genome research [18–21]. The thickness and composition of the spore wall of microsporidia (exospore, endospore, and plasma membrane) complicate the introduction of exogenous genes into microsporidia by currently available technologies, including homologous recombination-based gene targeting, gene transposition, and CRISPR/Cas9 gene editing [5, 22, 23]. At present, the common methods used to explain microsporidia functional genes and the infection mechanism include chemical methods such as fluorescent probe-labeling, biotin-labeling, antibody-labeling, or chemical reagent fluorescent brightener 28 (FB28), and 4′,6-diamidino-2-phenylindole (DAPI) nuclear dye [24–27]. Furthermore, although it has been reported that RNA interference (RNAi) can inhibit microsporidia gene expression, the interference efficiency is not high, and it is difficult to carry out systematic gene function research [28, 29]. Therefore, a method for introducing foreign genes into microsporidia is urgently needed to provide a basis for gene function research, prevention, and control of microsporidia.

Electro-transformation is an effective artificial transformation method that introduces natural or recombinant foreign protein-encoding genes into microorganisms for overexpression or homologous recombination for gene knockout or knock-in [30, 31]. It is widely used in the directional transformation of fungi and bacterial strains [32, 33]. Since 1993, foreign gene vectors have been introduced by electro-transformation into *Toxoplasma gondii* for stable and efficient expression, and then into *Plasmodium falciparum*, *Cryptosporidium parvum*, and *Eimeria intestinalis* [34–36]. The transformation efficiency of electro-transformation is affected by the growth stage of the parasite, components of the shock buffer, shock voltage, pulse time, electric field strength, resistance, plasmid DNA concentration, components of resuscitation medium, and the resuscitation time [32, 37]. At present, there is still no report of the introduction of foreign genes in microsporidia by electric transformation. Therefore, the establishment of such a method is crucial to providing key technical means for analyzing the mechanisms underlying the infection of microsporidia and the interaction between the pathogen and the host.

Microsporidia species have the smallest known eukaryotic genome. Such compactness needs to be investigated and may provide an experimental advantage [20]. Based on the previous experience of successful in vivo transformation in parasites, when designing a successful in vivo gene transformation method of *N. bombycis*, we first analyzed the efficiency of fluorescent protein expression by the original *Bombyx mori* A3 cytoplasmic actin promoter (A3) and *Orgyia pseudotsugata* multiple nucleocapsid nuclear polyhedrosis virus immediate-early 2 gene (OpIE2) promoter. Then, the *N. bombycis* transduction efficiency was explored according to the previously reported electro-transformation strategy. Finally, based on the analysis of the life history of *N. bombycis*, we discuss the best conditions for the electro-transformation of foreign genes into *N. bombycis*. The successful introduction of a fluorescent protein gene by electro-transformation undoubtedly allows foreign gene recombination, gene fusion expression, nonhomologous end joining, and gene editing in the *N. bombycis* genome.

## Methods

### Cells and *N. bombycis*

*Bombyx mori* embryo (BmE-SWU1) and *Bombyx mori* ovary (BmN-SWU1) cells were established and preserved in our laboratory [38, 39]. In addition, the *N. bombycis* CQ1 [no.: CVCC3088(L)] was isolated and preserved in our laboratory.

### Vector construction

The plasmid pBac-A3^prm^-enhanced green fluorescent protein (EGFP) and pIZ-OpIE2^prm^-Discosoma sp. red fluorescent protein (DsRed) was constructed by our laboratory. Briefly, the DsRed fragments were amplified using the primers DsRed-F: CCCAAGCTTATGGCCTCCTCCGAGAACGT and DsRed-R: CGGGGTACCCAGGAACAGGTGGTGGCGG. Subsequently, the polymerase chain reaction (PCR) fragments were connected to a pIZ-V5/His vector to generate pIZ-OpIE2^prm^-DsRed. The plasmids pBac-A3^prm^-EGFP were constructed using the *B. mori* cytoplasmic actin gene BmA3 promoter and the enhanced green fluorescent protein (EGFP) sequence, in addition to the SV40 polyadenylation signal sequence as described previously [40].

### Immunofluorescence

The microsporidia localization at different infection times was analyzed by immunofluorescence after infection of BmN-SWU1 and BmE-SWU1 cells with *N. bombycis*. Briefly, the sample was washed with phosphate-buffered saline (PBS), and 500 µl of 4% paraformaldehyde was then added to each well and left at room temperature for 15 min. After washing with PBS, 500 µl of 1% Triton X-100 was added and the sample was left at room temperature for 10 min, then incubated at 37 °C for 1 h. Finally, the samples were incubated with anti-PTP2 antibody (1:200, rabbit), incubated at 37 °C for 1 h, and then incubated with Alexa 488-conjugated goat anti-rabbit secondary antibody (1:1000) at 37 °C for 1 h. The nuclei-stained microsporidia were incubated with FB28 at 37 °C for 1 h. An Olympus FV3000 scanning electron microscope was used for image acquisition. Excitation wavelengths of 405, 488, and 561 nm were used for all the images.

### Electroporation of *N. bombycis*

Four hundred microliters of 1 M sorbitol and 5 µg of plasmid were added to a solution containing 1 × 10^7^ spores, then resuspended and mixed evenly, precooled on ice for 10 min, transferred to a precooled electrode cup, and left there for 5 min. The electric shock parameters were set, the shock was applied, the sample was left standing for 3 min and then washed, and BmN-SWU1 and BmE-SWU1 cells were then infected after 0.1 M KOH treatment**.** The electroporation system (CUY21EDIT II) was purchased from BEX Japan.

### Electroporation of cells

The BmN-SWU1 and BmE-SWU1 cells were collected in a 5 ml centrifuge tube and centrifuged at 800×*g* for 5 min, and the supernatant was removed. Next, 3 ml Opti-MEM (minimal essential medium) was added, the cells were centrifuged for 800×*g* for 5 min, and the supernatant was removed. Then, 25 µl of medium (cell content ≥ 1 × 10^6^) and 5 µg of plasmid were added, and the mix was transferred to a 2 mm electrode cup. Finally, the electro-transformation parameters were set, and the cells were electroporated and left to recover for 1 min. After electroporation, 200 µl of Opti-MEM medium were added to the electrode cup, and the cell suspension was transferred to 24-well plates. The cells were cultured at 27 °C, and the expression of the transformation plasmid was observed for 24 h.

### Statistical analysis

All of the statistical analysis was performed using Student’s *t*-test in GraphPad Prism 6 (http://www.graphpad.com) (GraphPad Software, Inc., San Diego, CA, USA). All the data are presented as the mean ± SD. Differences between groups were considered significant at the following significance levels, **P < 0.01.

## Results

### Plasmid transfection into *N. bombycis*-infected cells

**Fig. 1:**
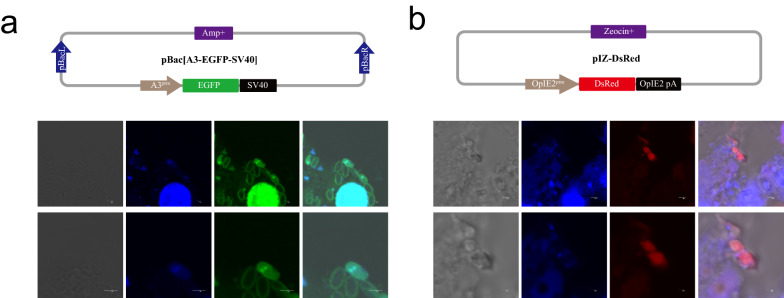
Transfection of plasmids into *N. bombycis*-infected cells. **a** Top: Schematic diagrams of the EGFP protein expression vector construction. In the plasmids used for transient expression in microsporidia, GFP was driven by the A3promoter. Bottom: Green fluorescence observation of liposome-transfected pBac-A3^prm^-EGFP plasmid into microsporidia-infected cells. Scale bar, 3 μm. **b** Top: Schematic diagrams of the DsRed protein expression vector construction. In the plasmids used for transient expression in microsporidia, DsRed was driven by the *OpIE2* promoter. Bottom: Red fluorescence observation of liposome-transfected pIZ-OpIE2^prm^-DsRed plasmid into microsporidia-infected cells

To analyze the expression of fluorescent protein after BmE-SWU1 cell infection with microsporidia, the *B. mori* actin 3 (A3) promoter and baculovirus *Orgyia pseudotsugata* multi nucleocapsid nuclear polyhedrosis virus (OpMNPV) OpIE2 promoter were selected to construct fluorescent protein expression vectors (Fig. 1a).

Since the best time for introducing a foreign gene into *N. bombycis* is unclear, multiple transfection at different times was performed to observe fluorescent protein expression. The silkworm cells were infected with microsporidia after transfection with fluorescent protein expression plasmid, and the fluorescent protein expression was observed at 96 h post–infection (p.i.). The expression of the red fluorescence and green fluorescence was clearly observable after transfection with pBac-A3^prm^-EGFP and pIZ-OpIE2^prm^-DsRed of *N. bombycis*-infected cells. In addition, the A3 and OpIE2 promoter expression vector could enter *N. bombycis* and express the fluorescent protein (Fig. 1b).

### Electro-transformation of fluorescent protein into *N. bombycis* spores

**Fig. 2:**
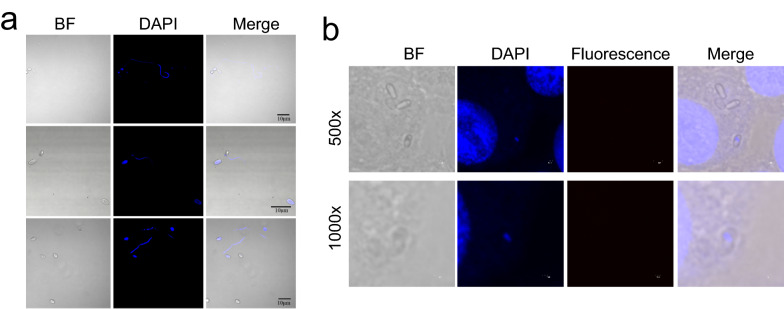
Electro-transformation of fluorescent protein into *N. bombycis*. **a** Artificial germination of *N. bombycis*. **b** Fluorescence analysis of fluorescent protein expression in BmN-SWU1 cell infection with *N. bombycis* after electro-transformation. Scale bar, 1 and 2 μm

To obtain high fluorescence intensity trace-*N. bombycis*, we attempted to electro-transform *N. bombycis* with a fluorescent protein-encoding plasmid. We analyzed the germination of *N. bombycis* with different electro-transformation parameters. Different electro-transformation conditions had some effects on the germination of *N. bombycis*, but all of them could normally germinate (Fig. 2a). Notably, the germination rate of *N. bombycis* was relatively high under the condition of 2.5 kV, 200 Ω, and 25 µF electric transformation (Table 1). Therefore, BmN-SWU1 cells were infected with *N. bombycis* under this electric-transformation condition (Fig. 2b). However, the expression of fluorescent protein could not be detected in *N. bombycis*. These results showed that the electro-transformation of *N. bombycis* could not introduce the plasmid into *N. bombycis*.

**Table 1:** Germination of microsporidia for different electroporation parameters

Germination	Parameter					
	4 kV 200 Ω 25 µF	3.5 kV 200 Ω 25 µF	3 kV 200 Ω 25 µF	2.5 kV 200 Ω 25 µF	2 kV 200 Ω 25 µF	1 kV 200 Ω 25 µF
0.1 M KOH/10 min	22%	15%	10%	30%	25%	21%
0.1 M KOH/20 min	23%	17%	10%	29%	25%	25%
0.1 M KOH/30 min	20%	17%	11%	32%	28%	22%
0.1 M DTT, 0.1 M sorbitol, 0.1 M SDS/10 min	25%	20%	10%	30%	28%	25%
0.5 M DTT, 0.5 M sorbitol, 0.5 M SDS/10 min	27%	20%	15%	32%	29%	28%
1 M DTT, 1 M sorbitol, 1 M SDS/10 min	25%	22%	11%	35%	32%	30%

### Tolerance of BmN-SWU1 and BmE-SWU1 cells to electro-transformation

Electro-transformation can electroporate the cell membrane, allowing the plasmid to enter the cell membrane instantaneously [33, 41]. Therefore, we carried out an electro-transformation experiment of microsporidia at the cellular level. The pIZ-OpIE2^prm^-DsRed plasmids were electro-transformed into the two cell lines. The results showed that under the same electro-transformation parameters, the BmE-SWU1 cell line had higher expression of the fluorescent protein than BmN-SWU1. Meanwhile, perforation voltage 100 V, pulse length 15 ms, pulse interval 10 ms, drive voltage 25 V, pulse length 60 ms, pulse interval 50 ms, and 10 cycles were the best electro-transformation conditions in perforation electrophoresis (Table 2).

**Table 2:** Parameter settings for *Bombyx mori* cell by electro-transformation

Numbers	Perforation pulse			Driving pulse				Transformation efficiency (%)
	Perforation voltage (V)	Pulse length (ms)	Pulse interval (ms)	Drive voltage (V)	Pulse length (ms)	Pulse interval (ms)	Cycles	
N1	100	20	10	20	50	50	10	4.09
N2	120	15	10	20	50	50	10	5.88
N3	140	10	10	20	50	50	10	2.80
N4	160	5	10	20	50	50	10	11.7
N5	100	15	10	25	60	50	10	6.59
N6	110	10	10	30	50	50	10	2.97
E1	100	20	10	20	50	50	10	6.96
E2	120	15	10	20	50	50	10	2.29
E3	140	10	10	20	50	50	10	2.43
E4	160	5	10	20	50	50	10	2.24
E5	100	15	10	25	60	50	10	12.02
E6	110	10	10	30	50	50	10	11.28

### Analysis of *N. bombycis* life history in BmN-SWU1 cells

**Fig. 3:**
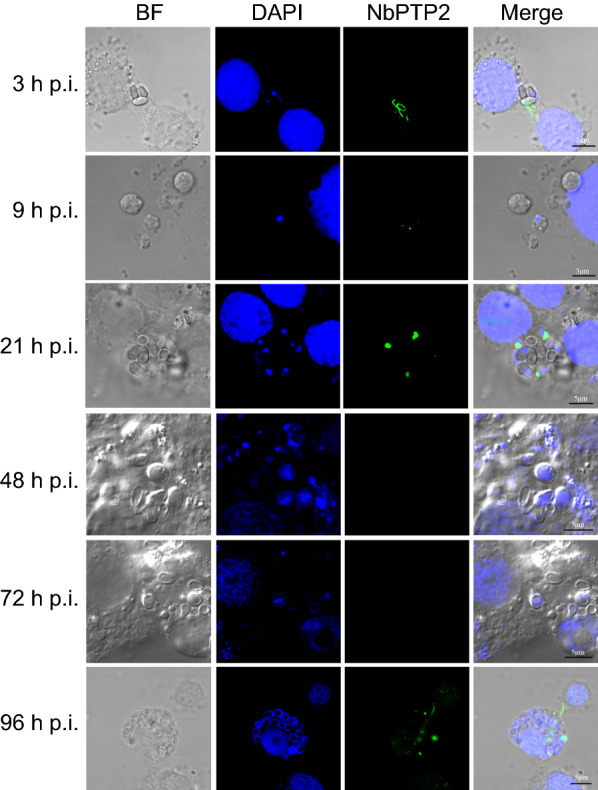
Analysis of *N. bombycis* life history in BmE-SWU1 cells. Immunofluorescence localization of NbPTP2 in *N. bombycis* infection. Green represents NbPTP2 protein expression; DAPI represents the nucleus of *B. mori* and *N. bombycis.* Scale bar, 5 μm

To determine the best electro-transformation period, we labeled the polar tube protein NbPTP2 and nuclear state during *N. bombycis* infection by immunofluorescence. *Nosema bombycis* ejected polar filaments to infect host cells, and NbPTP2 could be labeled after 3 h p.i. At 9–21 h p.i., the period of schizont *N. bombycis *with obvious membranc lysis could be observed (Fig. 3). At 48–72 h p.i., the form of binuclear and polynuclear of *N. bombyxcis* were stained by DAPI.  There was no sporoblast during at this time. It is speculated that the sporoblast began to develop 48–72 h p.i. At 96 h p.i., the spores entered host cells by ejecting polar filaments (Fig. 3). These results showed that 3–72 h p.i. is the critical period for *N. bombycis* proliferation, and an important period of electrical transformation.

### Transfection of *N. bombycis* for stable expression of fluorescent protein

**Fig. 4:**
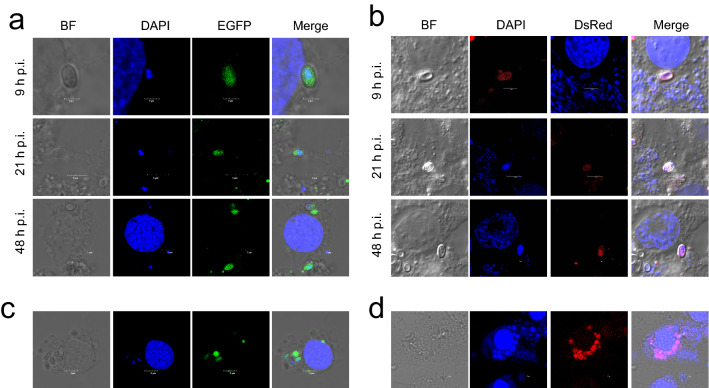
Transfection of the *N. bombycis* for stable expression of a fluorescent protein. Fluorescence analysis of *N. bombycis*-infected cells electro-transformed with plasmids. **a** Green represents green fluorescent protein expression; **b** Red represents red fluorescent protein expression. Scale bar, 3 μm. **c** Green represents green fluorescent protein expression. **d** Red represents red fluorescent protein expression. DAPI represents the nucleus of *B. mori* and *N. bombycis.* Scale bar, 3 μm

Based on the life history of *N. bombycis* infections, we selected 9 h, 21 h, and 48 h after infection for the electro-transformation experiment. We introduced pBac-A3^prm^-EGFP and pIZ-OpIE2^prm^-DsRed plasmids into the BmE-SWU1 cell line by electro-transformation. The green fluorescence proteins could be detected at 9 h p.i., 21 h p.i., and 48 h p.i., which indicates that the best period for foreign gene introduction is in the sporoplasm and sporogony stage of *N. bombycis* (Fig. 4a and b). It is speculated that *N. bombycis* has only one plasma membrane in the intracellular stage at 9–48 h p.i. Therefore, the foreign gene could easily enter *N. bombycis* by electro-transformation. Fluorescence observation also showed that regardless of the period of electro-transformation, the fluorescence signal could be detected at 84 h p.i. We further analyzed whether *N. bombycis* with fluorescent proteins could proliferate in BmE-SWU1 cells. The results showed that the fluorescence signal of several spores could be detected in a single image (Fig. 4c and d).

## Discussion

Microsporidia, a large class of unicellular eukaryotes with typical obligate intracellular parasitism, have been identified in more than 200 genera and 1500 species [42]. However, up to now, there have been no reports of gene mutation, genome deletion, or fusion expression of foreign genes in microsporidia, which seriously affects the interpretation of the microsporidian development cycle and the analysis of the infection mechanism. To overcome these challenges, in this study we successfully introduced genes encoding foreign fluorescent proteins into microsporidia by electro-transformation, and systematically analyzed the electro-transformation conditions and best opportunity for foreign gene introduction.

**Fig. 5:**
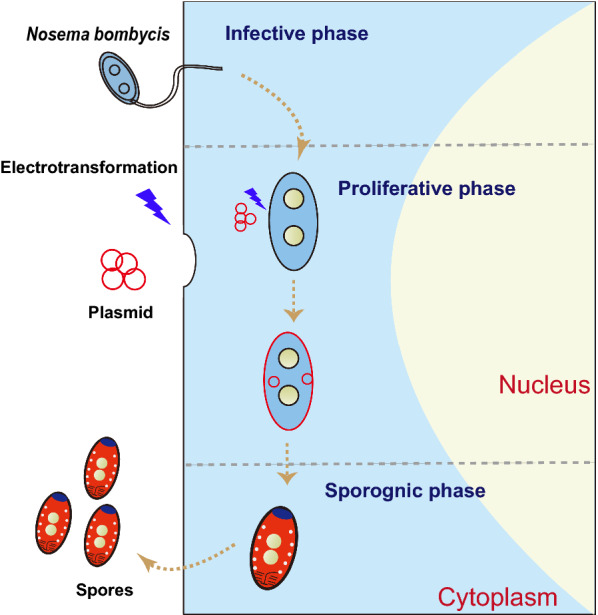
Schematic diagram illustrating the high-efficiency transformation of fluorescent proteins into *N. bombycis*

Microsporidia are unique intracellular parasites. The introduction of foreign genes into infected BmN-SWU1 through non-transposon vectors and the attempt to culture *N. bombycis* in vitro could not efficiently introduce foreign genes into the parasite [14, 43]. As an important transformation technology in molecular biology, electro-transformation has the advantages of a wide application range, high transformation efficiency, and simple operation [31–33]. It can be applied to microbial genetic engineering, mutation breeding, exogenous expression of related proteins, and directional transformation of strains [37, 39]. Therefore, this study attempted to introduce foreign genes into microsporidia through electro-transformation. We first tried direct electro-transformation of the *N. bombycis*, but no stable expression of foreign genes was reached. Given what is known about electrical transformation in *Plasmodium* and *Cryptosporidium*, we speculate that failure to transform the germinated microsporidia may be because, during this period, the spore protoplasm is not completely exposed to the culture medium, and the culture medium is not suitable for *N. bombycis* (Fig. 2) [35, 44, 45]. Exploring the electro-transformation methods in *N. bombycis* and the culture conditions is suitable for the proliferation of *N. bombycis* [45]. Therefore, we further analyzed the intracellular electro-transformation at different stages after infecting cells with *N. bombycis* and determined that the schizogony of *N. bombycis* was the key period for electro-transformation (Fig. 3). These results verified the electro-transformation conditions of microsporidia, which proved that changing the permeability level of the membrane could transform foreign genes more effectively and confirmed that foreign genes could not be effectively introduced during spore germination and mature spore morphology (Fig. 5).

Expression of fluorescent marker genes, homologous repair, gene design, anti-drug screening, gene knockout, and CRISPR library screening have been widely used in *Toxoplasma gondii* after transient transformation and expression of foreign genes based on electro-transformation [34, 46–48]. Therefore, based on the electro-transformation method, we can carry out further studies in microsporidia, as described in the following three examples: (1) According to the previous studies on methionine amino-peptidase (MetAP2)-resistant fumagillin, we could express fluorescent protein fusion resistance markers to screen the strains stably expressing the fluorescent proteins [45, 49]. (2) A long terminal direct repeat retrotransposon (LTR) transposon and a Tc1-like DNA transposon have been reported in the *N. bombycis* genome, and PiggyBac transposon could also be transposed in the genome of *P. falciparum* [45, 50–52]. Therefore, we could combine fluorescent protein, resistance marker, and transposon to transform foreign genes into the *N. bombycis* genome through electrical transformation. (3) CRISPR/Cas9 genome editing techniques are a powerful tool developed for many intracellular parasites. It is not difficult to imagine that genetic manipulation mediated by CRISPR will also be applied to the genome of microsporidia parasites as these techniques improve [46, 47]. It was previously reported that after inducing spore germination in vitro, the sporoplasm of *N. bombycis* was successfully isolated and identified [53, 54]. Combined with electro-transformation technology, it may be more efficient to introduce foreign genes in *N. bombycis*, which will enable rapid development in the genetic operation of *N. bombycis*.

## Conclusions

In conclusion, we systematically analyzed a method for successfully transforming exogenous genes into *N. bombycis*. In this study, we determined the conditions of punctured voltage, pulse length, and pulse interval, and we found that the schizogony period of *N. bombycis* is the best for a successful transformation. These results provide important insights for the development and application of *N. bombycis* genetic modification techniques.

## Data Availability

Data supporting the results of this study are included within the article. All data and materials are fully available without restriction upon reasonable request.

## Abbreviations

A3: *Bombyx mori* A3 cytoplasmic actin promoter
OpIE2: *Orgyia pseudotsugata* multiple nucleocapsid nuclear polyhedrosis virus immediate-early 2 gene
CRISPR/Cas9: Clustered regularly interspaced short palindromic repeats/CRISPR-associated 9
kV: Kilovolt
Ω: Ohms
µF: Microfarad
BmE-SWU1: *Bombyx mori* embryo Southwest University 1
BmN-SWU1: *Bombyx mori* ovary Southwest University 1
DNA: Deoxyribonucleic acid
EGFP: Enhanced green fluorescent protein
DsRed: *Discosoma *sp. red fluorescent protein
PBS: Phosphate-buffered saline
